# Enlarged Prostate
*A Paper read at a Meeting of the Bristol Medico-Chirurgical Society on 14th December, 1932.


**Published:** 1933

**Authors:** A. Rendle Short

**Affiliations:** Professor of Surgery in the University of Bristol; Surgeon to the Bristol Royal Infirmary


### ENLARGED PROSTATE.*

BY

A. Rendle Short, M.D., B.S., F.R.C.S.,

*Professor of Surgery in the University of Bristol; Surgeon to the Bristol Royal Infirmary.*

---

Enlarged prostate is a common disease. Most doctors probably see a case or two in the course of a year's work. At the Bristol Royal Infirmary one often has three or four cases side by side. It is no respecter of persons; patients who have lived a blameless life both morally and medically are by no means exempt. It is a condition which has been responsible in times past for an immense amount of suffering, and has added grievously to the burdens of old age. Some famous men have been its victims, including Dr. Ken, who was made Bishop of Bath and Wells in the reign of Charles II, was sent to the Tower with six other bishops by James II, and wrote a number of well-known hymns. In his old age, which was spent partly at Naish House, near Failand, and partly at Longleat, he suffered severely from the symptoms of enlarged prostate. Before surgery came into its own for this condition there was no resource except a catheter life, and this was usually a miserable existence. I well remember one of my patients, himself a sufferer, telling how he had to minister to his old father for about two years, and the pain and dismay which resulted.

Another reason why I have chosen this subject

* A Paper read at a Meeting of the Bristol Medico-Chirurgical Society on 14th December, 1932.

for consideration this evening is that it is becoming clear that a number of somewhat different anatomical conditions may be responsible for a patient's symptoms, and the signs and treatment are by no means always the same. Also, there are certain problems connected with the method of operating and after-treatment about which there is plenty of room for difference of opinion. The earliest age at which enlarged prostate has to be considered as a probable cause of symptoms is somewhere about 48 ; my youngest patient was 49. It is not common under 60 ; on the other hand, it may persecute patients to an extreme old age, and one fairly often has to operate on octogenarians ; my oldest was 85 ; he did excellently.

It is worth while to recognize the following anatomical conditions, all of which may be classed under the general heading of enlarged prostate :—

(1) General enlargement of the whole prostate. This bulges up into the bladder and back into the rectum and may reach a great size. It is often seen in very old men.

(2) General enlargement of the prostate composed of rounded nodules. This produces much the same signs and symptoms as the above.

(3) So called "middle lobe" cases. There is not much general enlargement, but the middle lobe stands up like a pillar behind the internal meatus.

(4) Prostatic bar. There is a hard ridge forming the posterior lip of the internal meatus, with little or no general enlargement of the prostate.

(5) Atrophic prostate. Small and hard, often associated with prostatic bar.

(6) Nodules in the floor of the prostatic urethra about the size of a pea, with little or no general enlargement.

(7) Cancer of the prostate. This is outside our subject.

It will readily be understood that all these differing types produce some variation in signs and symptoms. They have in common a progressing tendency, and the patient is likely to get steadily worse. In some cases the nodules in the prostate which were producing the symptoms are innocent for a time but develop malignant disease at a later stage.

In general, the first symptom of enlarged prostate is frequency of micturition, which for some unexplained reason is worse at night. The loss of sleep may be one of the most serious parts of the patient's disabilities. The cause of the frequency is not entirely easy to explain; probably the sphincter at the vesical orifice is impeded in its work by prostatic tissue enclosed within its circle, and thus readily allows a few drops of urine to trickle through into the urethra and start the reflex.

The passage of urine becomes more and more difficult, with straining and dribbling, and eventually goes on to acute retention. This is often precipitated by some indiscretion in drinking, or by the patient holding the water too long. Sometimes, after a few days of catheter treatment, the power of passing water is recovered for a time, but if this recovery does not take place within three or four days it is not likely that the patient will be able to function again without an operation.

From this standard picture there are many variations. Cases with a prostatic bar, or nodules in the urethra, and the atrophic prostates, will probably escape retention though they may suffer badly from frequency. In other cases the bladder gradually distends until the patient is only passing a few ounces every hour or so and retaining a pint or more of residual urine, so that the bladder is always to be felt reaching up to the umbilicus. They may go on for months in this condition. In other cases, and especially if the patient has been catheterized, his sufferings are increased by the advent of cystitis, which in its turn may go on to the formation of a stone. Bleeding after catheterization is common, and in a few cases it may occur spontaneously. Sometimes we are told that there has been no frequency prior to the onset of retention ; this may occasionally be a fault of memory, but in other cases it appears to be true.

Pain is not prominent at first, but when there is great straining, or retention, or cystitis, or stone, it becomes distressing. The straining not infrequently gives rise to rectal prolapse or to piles, or there may be partial incontinence of fæces. Eventually the general health begins to fail. Sometimes high blood-pressure appears to be due in part to prostatic obstruction. I have seen chronic bronchitis cured by prostatectomy. But, as a rule, the symptoms are those of chronic uræmia ; loss of appetite, a tendency to catheter fever, urinous breath, dry, furred tongue, twitching, drowsiness and the like. It is of great importance to remember that a patient's kidneys may be gravely impaired, and yet the mental and physical health appear to be normal.

The diagnosis of enlarged prostate ought not to be difficult, but it should be realized that the fact that no enlargement of the prostate can be felt *per rectum* is no proof at all that the gland is not the cause of the symptoms. The generally enlarged prostate does bulge into the rectum, but the enlarged middle lobe, the prostatic bar, the atrophic prostate, and the cases with a nodule in the urethra produce nothing that can be felt by the examining finger. The rectal examination should not be made when the bladder is full, or the prostate will be thought to be enlarged when it is nothing of the sort. In practice, therefore, one often has to make the diagnosis on symptoms, but in doing so, there are several conditions which must be thought of and excluded. I have been deceived once or twice by each of the following :—

*Stricture of the urethra* causes obstruction, but not as a rule frequency, and a catheter fails to pass.

*Stone in the bladder* may be detected by the metal sound, a skiagram, or cystoscopy.

*Cancer of the bladder* may mimic enlarged prostate very closely, and can only be recognized by the cystoscope.

To mistake vesical calculus, or cancer of the bladder, for enlarged prostate is not of very great moment, as the treatment advised will probably be surgical.

*Simple cystitis* is occasionally seen in old men, coming on without obvious cause, and to be cleared up by bladder washes and urinary antiseptics. It would be a serious mistake to open a bladder in such a case. Fortunately, pus in the urine is usually a late, not early, association with an enlarged prostate.

Three or four times I have had a patient come up for retention of urine and complete intestinal obstruction. In the first case I was misguided enough to perform colotomy, only to find that the cause of the obstruction was a full bladder impacted in the pelvis.

Cancer of the prostate is found in about one case in five with prostatic symptoms. It cannot be distinguished from innocent enlargement on the history, but the rectal examination shows stony hardness, fixity, and perhaps extension of the growth to the rectal wall or the base of the bladder.

General enlargement of the prostate and middle lobe cases can be diagnosed by a cystoscopic examination, or by passing a catheter after the patient has, as he thinks, emptied the bladder, and finding residual urine. There are, however, objections to instrumentation in many cases. It frightens and distresses the patient, and may lead to cystitis. If the diagnosis is reasonably certain, I do not usually pass any instrument until the patient is under the anæsthetic for the operation.

Before we are in a position to advise treatment it is essential to arrive at an accurate opinion as to the function of the kidneys. Renal efficiency or inefficiency is the key to the prognosis. It must never be forgotten that *symptoms* of chronic uræmia are only seen when the kidneys are desperately diseased; the patient may appear to be in good general health when his renal functions are so bankrupt that an operation to remove the prostate straight away would kill him. We have, therefore, to resort to special tests to determine renal function.

(*a*) *Polyuria and the Specific Gravity Test.* According to the modern theory the glomeruli of the kidney filter out from the blood vast quantities of plasma minus proteins, while the renal-tubule-cells re-absorb most of the water, glucose and salts, but let pass the urea, uric acid, sulphates, etc. If on account of back pressure the tubule cells are damaged they fail to absorb, and the urine is dilute and excessive because they cannot do normally active work against the osmotic pressure exerted by the urea. If the daily output of urine is over 60 ounces, and the specific gravity is low, the kidneys are damaged. In these cases the normal daily variations in the specific gravity of the urine are flattened out, and the figure remains about the same night and day.

(*b*) *The Blood Urea Test.* A few c.c. of blood are sufficient for the estimation. The normal lies between 20 and 40 mgrm. (that is, between 0·02 and 0·04 per cent.). 80 mgrm. would mean very seriously damaged kidneys.

(*c*) *The Urea Concentration Test.* The patient is given 15 grammes of urea in 4 ounces of water by mouth, and fluids are restricted. The urine is collected one, two, and three hours after, and the concentration of urea estimated. This can be done in a few minutes, without any mathematical calculations, by means of the Doremus ureameter. Good kidneys will concentrate to 3 per cent.; anything under 1 per cent. means very serious damage. Opinion differs as to which of these three tests is the more reliable. Personally, I pin my faith to the urea concentration test. It certainly has the advantage of simplicity.

The treatment of enlarged prostate may be considered under the headings of non-operative and operative.

1. Non-operative treatment is always welcomed by the patient, but has a strictly limited field. It may be classified under three headings: catheter life, X-ray treatment, and "masterly inactivity." To take the last first, a policy of inactivity may be followed, because the patient is too bad, or because he is not bad enough. If on account of cardiac trouble, or severe bronchitis, or drowsiness due to uræmia, operation would evidently be hopeless, and the patient is not in pain nor completely obstructed, it is best to do nothing. Such cases nowadays are seldom seen. Again, there are men who have enlarged prostate and have to pass water once or twice during the night, but there is no straining or retention and no pain. Some of these may be let alone for a time, at any rate. But this is a policy which is only safe in a few. I well remember one patient whom I allowed to pay a visit to America before having his operation, because it seemed an early and favourable case. When he came back the kidneys were gravely affected, and he died of gangrene of the palate, no doubt uræmic in origin. That inactivity was anything but masterly. To postpone wisely we must investigate carefully, and be sure that there is no cancer of the bladder masquerading as enlarged prostate, that the renal functions are not impaired, and that residual urine is not above an ounce or two. Of course, in a man of seventy-five or eighty one ought not to advise surgical treatment unless the patient is already suffering considerable inconvenience, but in a man under sixty it must be remembered that we are dealing with a progressive disease, and it is better to operate while the going is good.

Catheter life is a miserable existence. A few men have bladders that will stand any amount of cystitis, and urethras like a tube of brass ; in days gone by these lived the catheter life fairly happily for years. The great majority are dead in six months. In practice it is seldom that the doctor can pass the catheter two or three times a day, so proper precautions are not taken, and septic infection carries the patient off.

X-ray treatment, in a few selected cases, has a definite value. The prostate must be generally enlarged ; the obstructive middle lobe cases, the prostatic bar, and the nodule in the urethra are not likely to be benefited. Retention or semi-retention must not be present, or valuable time may be wasted and the kidneys allowed to fall into bankruptcy before the inevitable operation is performed. Men past eighty are the most suitable subjects, and I have known one or two considerably improved.

2. Operative treatment is necessary in the great majority of cases, and can usually be relied on, if the patient survives, to give great relief; it often makes old men young again.

The routine treatment is prostatectomy, but there are several variations possible, including the punch operation, the diathermy-punch, partial prostatectomy, and permanent suprapubic drainage. Operation having been decided upon, it is necessary to consider whether it shall be a one-stage operation, or whether to drain the bladder first, wait a while, and then remove the prostate. Here opinion differs. Some surgeons always do a one-stage, others invariably practise the two-stage method. Personally, I take each case on its merits. In my last 118 cases there were 63 done in one stage and 55 in two. There were 41 others treated by suprapubic drainage which never came to prostatectomy. My rule is to use a two-stage procedure if the urea concentration test is below 1·8 per cent., and always if an acute retention is present, or the patient habitually carries a bladder up to the umbilicus. I can remember when for lack of this precaution the mortality in Bristol used to approach 50 per cent.

In the two-stage method, at the first operation, a large tube is inserted into the bladder above the pubes if the time interval is to be only a few weeks, or a Pezzer tube if it is to be long. If the bladder is up to the umbilicus it must be emptied very gradually, as by tying a No. 2 catheter in the urethra for twenty-four hours. Rapid emptying may be fatal from anuria; in these cases the valvular action of the ureters is often incompetent, and drawing off the water suddenly reduces the pressure, not only in the bladder but also in the kidneys, which is ruinous to the tubule cells.

The interval between the first and second stages is normally two or three weeks, but in heavy risk cases, with nearly bankrupt kidneys and urea concentration test under 1 per cent., it should extend to months. I have been able to bring through some very bad starters in this way, including a man whose concentration test was as low as 0·5, where we waited four months. The drainage allows time for the kidneys to recover and for sepsis to run itself out, aided by bladder washes, and pyridium by mouth. Pyridium is much more effectual than hexamine.

I now perform prostatectomy by the open (Thomson Walker) method, with a long incision; the prostate can usually be shelled out without a finger in the rectum. A great advantage of this over the old method with a small incision is the diminution of post-operative spasm-pain. A large tube is left in the bladder for two days, and a catheter tied in the urethra for four days to allow irrigation. Sometimes, without taking too much time over it, we can, by suturing, get the cavity left by the prostate sufficiently clean and dry not to need a pack. More often I think it is better not to prolong the operation, but to put in a pack soaked in mercurochrome. It not only checks bleeding, but also prevents the formation of a septum across the cavity. The pack is taken out, under gas, on the second day. One should have another ready to go in again if necessary; on three occasions I have had severe hæmorrhage. Some surgeons never use a pack. I envy their skill, or their boldness. Some surgeons use a Pilcher bag in the cavity, but my experience of it has been disastrous. If there is no pack, one can profitably use continuous aspiration for forty-eight hours, putting a suction-tube down inside the big rubber suprapubic drain. This avoids the need for a Hamilton Irving apparatus, and prevents peri-vesical septic infection. If there is a pack, or if no suction apparatus is available, continuous irrigation through the catheter for forty-eight hours, followed by irrigation twice a day, is valuable. A Hamilton Irving apparatus must be worn. It is a good thing to block one of the holes, and let the tube from the other drain to a bottle over the side of the bed, out of the patient's way. In my experience the first normal micturition is on the fourteenth to the seventeenth day.

Since the end of the Great War I have removed the prostate in 118 cases, 58 at the Infirmary and 60 in private. Amongst the private cases the mortality in the nursing home was 11·6 per cent.; in the total series of 118 it was 13·5 per cent. For the sake of comparison some recent figures are shown in the following table :—

*Mortality of Suprapubic Prostatectomy.**

| | No. of cases. | Died per cent. |
|---|---|---|
| Thomson Walker (private cases) | 472 | 5·2 |
| St. Peter's Hospital .. .. .. | 2,691 | 9·9 |
| Eleven general hospitals .. .. | 3,451 | 19·5 |
| Present series (private cases) .. | 60 | 11·6 |
| ,, ,, (all cases) .. .. | 118 | 13·5 |

The variation in the figures is remarkably wide and calls for some notice. No one would question the superior skill and vast experience of Sir John Thomson Walker and some other specialists in urology. But to be fair to the general surgeons, one must call

* Sir J. Thomson Walker, Lettsomian Lecture, *Lancet*, 1930, i., 1,163, 1,219, 1,273.

attention to the fact that in a series of 274 of his cases, in 70 per cent. the urea concentration was over 2 per cent., whereas in my series it was over 2 in only 35 per cent. That is to say, he had 30 per cent. of heavy-risk cases; we had 65 per cent. Evidently the specialist in urology gets a large proportion of favourable early cases.

A few words as to complications and causes of death. The main cause of death is uræmia from renal insufficiency, with or without septic infection. Shock, hæmorrhage, sudden cardiac failure, pneumonia, diarrhœa, thrombosis of the iliac veins, and gangrene of the throat have each carried off one or two of my fatal cases. The uræmic deaths could sometimes have been avoided by a two-stage operation. The less serious complications have been partial intestinal obstruction, septic epididymitis, and septum formation. Partial intestinal obstruction comes on within a day or two of the operation, and is, in my opinion, due to perivesical cellulitis, or abscess formation, between the bladder and the rectum. In two of my cases it cured itself by discharging pus into the rectum; in others there was a mucoid discharge *per anum*. Epididymitis comes on about a fortnight after operation, and is heralded by a rise of temperature. It usually subsides in a few days; in one of my cases an abscess formed and had to be opened. Some surgeons cut the vas deferens to prevent this complication. Septum formation is a great nuisance when it occurs, and makes itself evident about the third week. The mucosa of the base of the bladder has united across the top of the cavity left by the prostate, with or without a small, unhealed orifice. The patient is late to start passing water, and has to strain; a catheter will not pass into the bladder, and the suprapubic fistula will not close. I have had three or four cases, and all amongst the small number in which a pack was not used. To avoid this trouble, if one does not wish to pack, it is recommended to sew down flaps of mucosa in the side of the cavity. If septum formation has taken place, there is nothing for it but to re-open and cut it away.

Finally, a few words may be said with regard to other operations besides prostatectomy. When the patient is a good starter it is more satisfactory to remove the gland, but in a heavy-risk case one may do less and get a good result with some types of the disease. The generally enlarged prostate does not lend itself to partial operations, but the thumb-like middle lobe, the prostatic bar, and the nodule in the urethra may be nipped away by means of Jessop's prostatic punch, with the bladder opened and the rest of the gland left behind. Once or twice I have given relief by shelling out an adenoma from the gland. At least two diathermy punches have been introduced. I have some experience of one invented by K. M. Walker, which is passed *per urethram* and can be used with or without an open bladder. There is a more recent one invented by Canny Ryall. These instruments have a distinct place in the treatment of heavy-risk cases with enlarged middle lobe or prostatic bar.

Lastly, the patient with grave cardiac disease or nearly hopeless kidneys, and more or less complete retention of urine, can be given great relief by some form of permanent suprapubic drainage, though there is no wholly satisfactory apparatus for the purpose. Pezzer's tube can be got to fit tightly and not leak even if the man leads an active life, but it needs to be changed every six weeks, which may necessitate an anæsthetic. A plain tube and flange works well sometimes if the patient is in bed, not otherwise. Arnold's three-way suprapubic box and urinal looks promising, but I have found it difficult to adjust satisfactorily.

In my post-war series of 159 cases, not including cancer of the prostate, which is outside the scope of the present article, 41 were treated by suprapubic drainage. In some of these it was intended, if all went well, to remove the prostate later; in others the drainage was to be permanent. Eleven died; the others were sent home still wearing a tube.

I fear there is but little reference in this paper to the writings of others. It was my purpose to place before you, not a summary of the literature, but my own experience since the end of the war.

